# Exposing Structural Variations in SARS-CoV-2 Evolution

**DOI:** 10.21203/rs.3.rs-800496/v1

**Published:** 2021-09-13

**Authors:** Jiaan Yang, Peng Zhang, Wen Xiang Cheng, Youyong Lu, Wu Gang, Gang Ren

**Affiliations:** Chinese Academy of Sciences; Chinese Academy of Sciences; Chinese Academy of Sciences; Peking University Cancer Hospital & Institute; Huazhong University of Science and Technology; Lawrence Berkeley National Laboratory

**Keywords:** Mutation, SARS-CoV-2, spike protein, physiochemical properties, folding conformation

## Abstract

The mutation of SARS-CoV-2 influences viral function as residue replacements affect both physiochemical properties and folding conformations. Although a large amount of data on SARS-CoV-2 is available, the investigation of how viral functions change in response to mutations is hampered by a lack of effective structural analysis. Here, we exploit advances in protein structure fingerprint technology to study the folding conformational changes induced by mutations. With the integration of both protein sequences and folding conformations and alignments of SARS-CoV to SARS-CoV-2, the UK variant and India variant, we found that structural variations in the spike protein at the binding interface interacting with ACE2 play a critical role in coronavirus entry into human cells. Additionally, the structural variations impact vaccine effectiveness and drug function over the course of SARS-CoV-2 evolution. The analysis of structural variations revealed how the coronavirus has gradually evolved in both structure and function and how the SARS-CoV-2 variants have contributed to more severe acute disease worldwide.

## Introduction

Severe acute respiratory syndrome coronavirus 2 (SARS-CoV-2) belongs to the coronavirus family, and it is an urgent need to explore the SARS-CoV-2 structure, function and activity.^1^ In particular, mutations in SARS-CoV-2 are considered a priority because of their potential to increase transmissibility and virulence while reducing the effectiveness of vaccines and impacting the development of medical treatments.^2,3^ Mutations that alter the protein sequence, including replacements or deletions of amino acid residues, may affect protein properties and folding conformations and result in changes to the biological functions of the virus.^4^ The interaction of the receptor-binding domain (RBD) of the spike protein of SARS-CoV-2 with angiotensin-converting enzyme 2 (ACE2) receptors is key for allowing the virus to enter human cells.^5,6^ Thus, mutations in the RBD directly influence disease development and epidemic spread.^7,8^

To date, over 3,000 SARS-CoV-2 sequences and nearly 800 spike protein 3D structural data sources are available in the National Center for Biotechnology Information (NCBI) database and Protein Data Bank (PDB). According to the COVID-19 Genomics UK (COG-UK) Consortium, more than 4,000 mutations have been detected in the spike protein alone,^9^ which provides sufficient data to investigate coronavirus mutations to understand changes in its physiochemical properties as well as folding conformations leading to virus evolution over time. With protein sequence alignment, the positions of replaced amino acid residues can be discovered, and the concomitant changes in physiochemical properties can be further probed.^9,10^ In addition to physiochemical properties, changes in the protein folding conformation also impact biological viral functions. For proteins with known 3D structures, the conformational differences caused by mutations can be roughly compared by structure superposition with root-mean-square deviation (RMSD) as a measurement.^11^ For proteins without known 3D structures, the protein structures first need to be predicted by computational dynamics simulations. However, for mutational differences, the reliability of the predicted protein structure remains a challenge even when using *ab initio* modeling methods.^12–14^ Thus, it is crucial that a new approach overcomes these barriers to studying structural mutations.

At this point, we propose using the protein structure fingerprint approach^15,16^ to analyze the changes in folding conformation caused by mutations. With protein structure fingerprints, the protein folding shape code (PFSC) provides an alphabetical string to completely describe the folding conformation for 3D protein structure. Additionally, according to the protein sequence, the protein folding variation matrix (PFVM) reveals the folding variations along the sequence and generates the possible folding conformations. Thus, the alignment of the protein sequence with the PFSC string can comprehensively expose the variations in both biological functions and folding conformations caused by mutations in SARS-CoV-2. Here, the structural variations in evolved coronavirus strains, from SARS-CoV to SARS-CoV-2, UK variant and India variant, are studied.

## Methods

### Structural bioinformation.

All protein structural data for SARS-CoV-2 were extracted from public databases. The sequences were obtained from the NCBI and UniProt databases, and protein 3D structures were obtained from the PDB. The cd21477 cluster was obtained from the NCBI Conserved Domain Database, which contains the protein structure with PDB ID 6ACC for the SARS-CoV spike protein released in August 2018 and the protein structure with PDB ID 6VSB for the SARS-CoV-2 spike protein released in February 2020. Then, the mutations between 6ACC and 6VSB were analyzed according to either protein 3D structures or sequences by protein structure fingerprint technology. Information on the UK variant and India variant of SARS-CoV-2 was obtained from Public Health England. Seven mutations in the spike protein were identified, and the variations in physiochemical properties and folding conformations were studied. The complexes of coronavirus with ACE2 were obtained from PDB; PBD ID 6ACG is the complex with SARS-CoV, and PBD ID 7A98 is the complex with SARS-CoV-2.

### Protein comparison.

The sequences of the spike protein between SARS-CoV and SARS-CoV-2 were aligned with the Clustal Omega program through UniProt and then compared according to their physiochemical properties. Discovery Studio (version 4.5) was used to generate 3D images of protein structures, and then the superimposition of protein 3D structures was performed. Furthermore, with protein structure fingerprint technology, the variations in protein folding conformations were exposed in detail.

### Protein structure fingerprint.

First, the complete folding space for a set of 5 successive points was mathematically covered by a set of folding shapes. Second, the possible folds of a fragment of 5 amino acids could be defined by the 27 protein folding shape code (PFSC) with alphabetical letters, as shown in Fig. 4. Third, any protein sequence has a protein folding variation matrix (PFVM), and any protein with a given 3D structure can be expressed by a PFSC string as a protein structure conformation. In the PFSC string, two PFSC letters next each other overlap by four amino acids; thus, a PFSC string represents the complete folding conformation of the 3D protein structure. It is significant that protein folding conformations as PFSC strings can be aligned for comparison. Therefore, the folding variations of SARS-CoV-2 as well as its mutations may be well analyzed by protein structure fingerprints.

### Software availability.

The protein structure fingerprint can be accessed on Website http://www.micropht.com.

## Results

The changes in both physiochemical properties and folding conformations of SARS-CoV-2 due to mutation are studied based on the protein 3D structures and sequences of the spike protein, and the interaction between coronavirus and ACE2 are a particular focus. The structural analysis covers coronavirus strains from early SARS-CoV to SARS-CoV-2, UK variant and recent India variant.

### Variations based on 3D structures.

Coronavirus spike proteins have an S1 subunit at the N-terminus (~700 amino acids) and anS2 subunit at the C-terminus (~600 amino acids). Analysis of many protein 3D structures confirmed that three S1/S2 heterodimers assembled to form a trimer spike protruding from the viral envelope.^17^The S1 subunit of the spike protein in SARS-CoV-2 is an envelope glycoprotein that plays the most important role in viral attachment, fusion, and entry into host cells, and is a major target for the development of neutralizing antibodies, inhibitors and vaccines. The S1 subunit contains a receptor-binding domain (RBD), and many studies have found that the RBD of the spike protein in SARS-CoV-2 strongly binds to human and bat angiotensin-converting enzyme 2 (ACE2) receptors.^18–20^ A set of sequences for the cluster of the SARS-CoV-like_Spike_S1_RBD subfamily (cd21477) that contains the conserved protein domain of the S1 RBD subfamily for SARS-CoV-like and SARS-CoV-2 spike proteins is available in the NCBI database. The sequences of the cd21477 cluster were aligned and are presented in Table 1, where the red color font indicates highly conserved fragments, blue indicates less conserved fragments and gray indicates unaligned fragments. It is not surprising that the mutations were most frequent on less conserved residues (blue font). Additionally, it is noted that some sequences have the given 3D structures in the PDB. The protein structure of the SARS-CoV spike protein, with PDB ID 6ACC, was released in August 2018;^21^ the protein structure of the SARS-CoV-2 spike protein, with PDB ID 6VSB, was released in February 2020.^17^ The residues that differ between 6ACC and 6VSB are marked in green. Some changes in the physiochemical properties of the protein based on the differences between 6ACC to 6VSB are summarized in Table 2, including hydrophobicity, negative or positive charge, polarity, size of side chain, aromatic and etc. The changes in the physiochemical properties are represented by a “+” sign for an increase in the property after mutation and a “−” sign for a decrease in the property after mutation.

**Figure 1:**
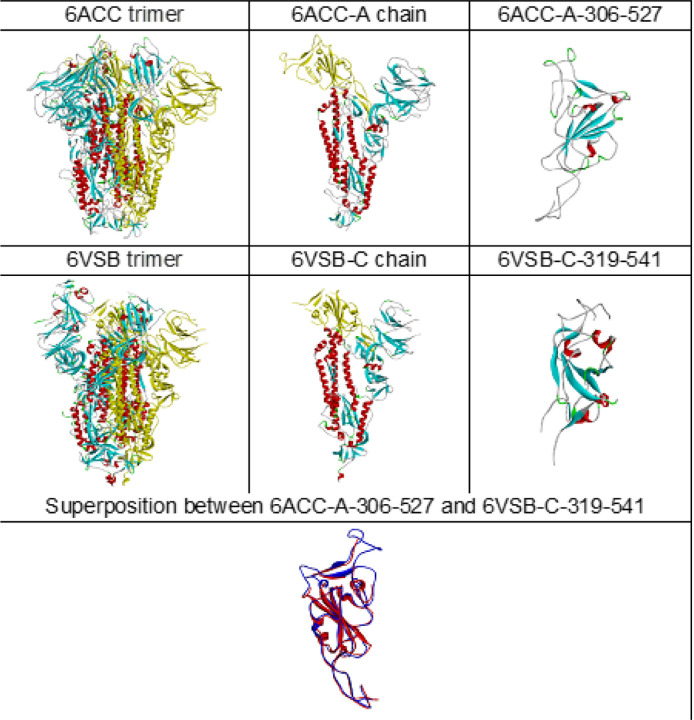
Comparison 3D structures between 6ACC and 6VSB. PDB ID 6ACC is the SARS-CoV spike protein, and PDB ID 6VSB is the SARS-CoV-2 spike protein. The protein 3D structural trimer, chain and domain fragment are displayed. The superposition of fragments between 6ACC-A-306-527 (blue) and 6VSB-C-319-541 (red) are shown at the bottom.

From the given structures of 6ACC and 6VSB, 3D images of folding conformations are compared and displayed in Fig. 1. The 3D structures directly provide a visualization to observe the protein structures, and the superposition allows a comparison of the structures. Although more than 30 mutations in the fragment between 6ACC-A-306-527 for SARS-CoV and 6VSB-C-319-541 for SARS-CoV-2 occurred, representing up to 37.5% residue replacement, the structure superposition showed that the folding conformations of 6ACC and 6VSB were still similar overall. It is difficult to distinguish the folding differences of spike proteins of SARS-CoV and SARS-CoV-2 based on the 3D structure only. With the protein folding shape code (PFSC), however, the differences in folding can be exposed. Any protein 3D structure can be converted into a PFSC description, which is an alphabetical string representing the continuous folding shape of each five-amino-acid in sequence. Thus, the folding conformations of 6ACC for SARS-CoV and 6VSB for SARS-CoV-2 can be compared by PFSC alignment and displayed in Table 3. In PFSC, generally, the red color indicates typical alpha helix, pink indicates alpha-like helix, blue indicates a typical beta strand, light blue indicates a beta-like strand, and black indicates an irregular fold. According tothe PFSC color notation, it is obvious that the secondary structural fragments are well aligned. For example, the fragments of alpha helices at 324–330 and 352–364 and the beta strands at 349–351 and 378–386 on 6ACC are aligned with the corresponding fragments in 6VSB. Also, the PFSC alignment exposes local folding comparison in detail, which the local folding similarity and differences between PFSC strings are indicated; “|” indicates an identical folding shape, “:” indicates a similar folding shape, and “.” indicates dissimilar folding. For example, the folding letters at 334, 335, 340, 341, 343, 370, 379 and 380 on 6ACC are different from 6VSB. Also, due to mutations, the adjustments of beta strand at 349–351 and 452–454 fragments on 6ACC are exposed. Thus, the PFSC revealed the changes in local folding shapes caused by the mutations.

### Variations based on sequences.

**Figure 2:**
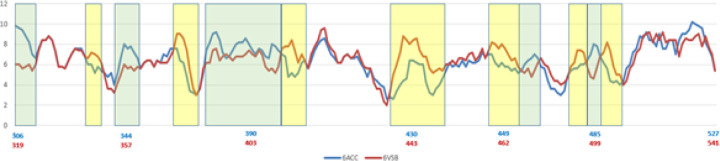
The numbers of folding variations in PFVM between 6ACC-A-306-527 for SARS-CoV and 6VSB-C-319-541. The horizontal coordinate is the sequence position, and the longitudinal coordinate is the number of folding shapes, i.e., number of PFSC letters. Yellow indicates the ranges with more variation in SARS-CoV, whereas green indicates more variation in SARS-CoV-2.

The variations of folding conformations for a protein based on sequence alone can be exposed by the PFVM. The PFVMs for sequences taken directly from the structures of 6ACC-A-306-527 for SARS-CoV and 6VSB-C-319-541 for SARS-CoV-2 separately are exhibited in Table 4. The PFSC letters in each column represent the folding variations of 5 successive amino acid residues in sequence while the favored folding shapes are ranked on top, and the numbers are different in each column. The PFVM exhibits the folding variations along the sequence. The number deviations of folding shapes along sequences in PFVM between 6ACC-A-306-527 for SARS-CoV and 6VSB-C-319-541 for SARS-CoV-2 are shown by the curves in Fig. 2, where the yellow and green blocks indicate the regions of fluctuation due to mutations. It was apparent that the mutations caused changes in folding flexibility; some fragments have the potential to be more flexible, and other fragments are more rigid. Thus, along the sequence from the N-terminus to the C-terminus, the variations in the folding conformation are well exposed.

The most likely conformations for a protein can be predicted from PFVM. Taking one letter from each column, a massive number of PFSC strings can be formed, and each string is one possible folding conformation. Although a large number of folding conformations exist, the letters on top of each column are directly constructed into one of the most likely conformations, which is named PFVM-01. This predicted conformation may be assessed by a given 3D protein structure through PFSC alignment. Two PFSC strings of the first row from Table 4 for 6ACC-A-306-527 and 6VSB-C-319-541 and two PFSC strings of their 3D structures from Table 3 are aligned in Table 5. The PFSC letters in red and pink colors represent alpha helices, those in blue and light blue represent beta strands and those in black represent irregular folding shapes. Overall, with observation, the secondary fragments are aligned, so the predicted folding conformations of PFVM-01 for SARS-CoV and SARS-CoV-2 are similar to the given 3D structures. Thus, the PFVM-01 as the most likely conformation, which is generated from PFVM, is a reliable prediction.

### Structure variations with virus evolution.

The sequence of the SARS-CoV spike protein (UniProtKB = P59594 (SPIKE_SARS)) was first determined in 2003.^22^ The sequence of the SARS-CoV-2 spike protein (UniProtKB = P0DTC2 (SPIKE_SARS2)) was determined in January 2020.^23^ After 17 years of evolution from SARS-CoV to SARS-CoV-2, the spike protein sequences are approximately 24% different. The UK variant is mutant of SARS-CoV-2 that was noted in November 2020 from a sample taken in the UK in September. SARS-CoV-2 infections in the UK increased because of one or more mutations in the virus spike protein. The lineage of the UK mutant B.1.1.7 VOC-202012/01 is taken from the Public Health England,^24^ which was reported on March 5, 2021, with seven mutations in the spike protein: E484K, N501Y, A570D, P681H, T716I, S982A and D1118H.^25^ Similar variants have also emerged in South Africa (lineage B.1.351) and Brazil (lineage P.1). Recently, India variant (lineage B.1.617.2) with mutations K417N, N440K, L452R, T478K and E484Q in the spike protein caused the epidemic to become severe. Thus, it is important to understand the effects of the mutations following virus evolution.

In order to study the mutations in SARS-CoV-2, a sequence of QTJ15692 (GenBank) was taken as the background reference, which was deposited in the NCBI database on April 2020 before the UK mutation. The mutations may cause changes in physicochemical functions as well as in folding conformations, which together impact biological functions. The changes in physiochemical properties, including hydrophobicity, negative or positive charge, polarity, residue size and aromaticity, are listed in the top rows of Table 6. For example, the mutation E484K changed a negative charge to a positive charge; A570D is a change from hydrophobic to negative, from non-charged to charged and from non-polar to polar; P681H is a change from hydrophobic to positive, from non-charged to charged and from non-polar to polar and an increase in the size of the side chain due to an aromatic moiety; T716I and S982A are changes from polar to hydrophobic. The changes in physiochemical properties caused by mutations are indicated in detail. Furthermore, the variations in local folding shapes may be revealed by PFVM because each PFSC letter in PFVM represents the folding shape of 5 successive amino acids in sequence. The PFVMs of seven regions for these related mutations are displayed in Table 7, which shows the folding variations before and after mutations. To compare each pair of PFVMs, the fluctuations in the number of folding shapes and the contributions of the alpha helix and beta strand are summed and listed in the bottom three rows in Table 6. It is apparent that the number of folding variations is reduced after mutation for seven regions, which indicates that the flexibilities are reduced. For the E484 mutation, the contribution of the alpha helix increased while that of the beta strand was reduced; for N501Y, the contribution of the alpha helix decreased while that of the beta strand increased; for D1118H, the factor of contribution of the alpha helix decreased while that of the beta strand increased. Therefore, the variations in both physiochemical properties and folding features for UK mutations of SARS-CoV-2 are well exposed.

### Mutations vs. ACE2 interaction.

**Figure 3:**
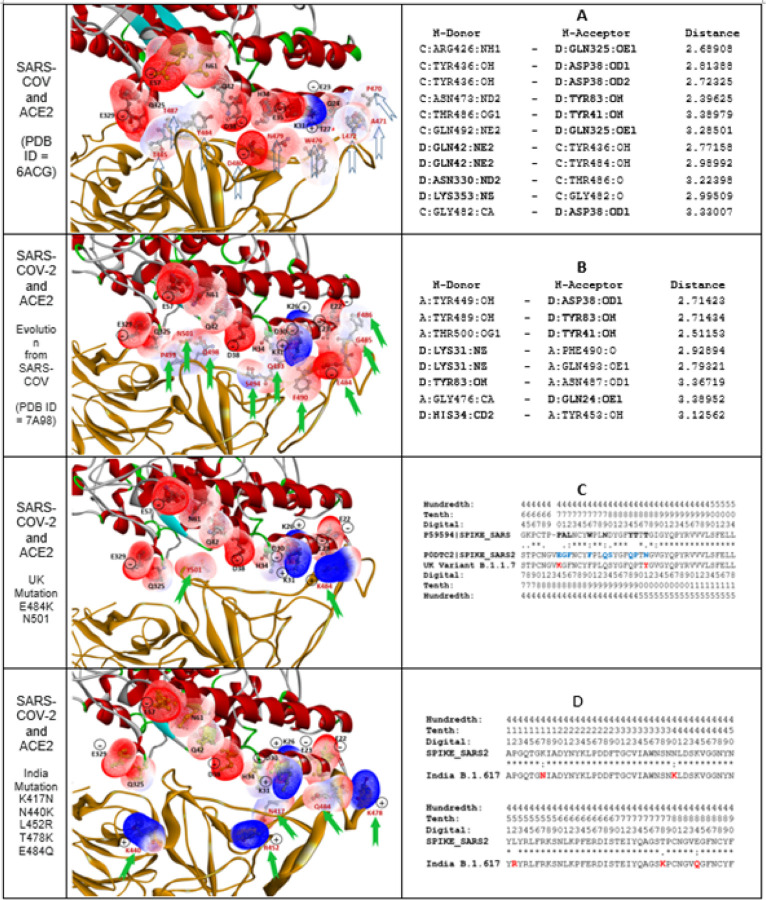
Mutations impact the interaction between SARS-CoV-2 and ACE2. The 3D images display the binding interface between the RBD of the SARS-CoV-2 spike protein and ACE2. The protein structure shown in brown color is SARS-CoV or SARS-CoV-2. The wire meshes represent the charge surfaces for residues involved in the interaction; red wire mesh indicates negative charge, and blue indicates positive charge. Row A shows the SARS-CoV structure and intermolecular H-bonds (PDB ID 6ACG); row B shows SARS-CoV-2 and H-bonds (PDB ID 7A98). Row C shows the structure of the UK variant, and row D shows the structure of the Indian variant, which were both obtained by computational modeling. The contributions of hydrogen bonds from ACE2 are marked by bold font. The arrows indicate the residues altered in viral evolution. The sequences of SARS-CoV (UniProtKB = P59594 (SPIKE_SARS)), SARS-CoV-2 (UniProtKB = P0DTC2 (SPIKE_SARS2)) and the UK variant and Indian variant are aligned and listed in row C and D, and the mutational residues are shown in colored bold font.

**Figure 4:**
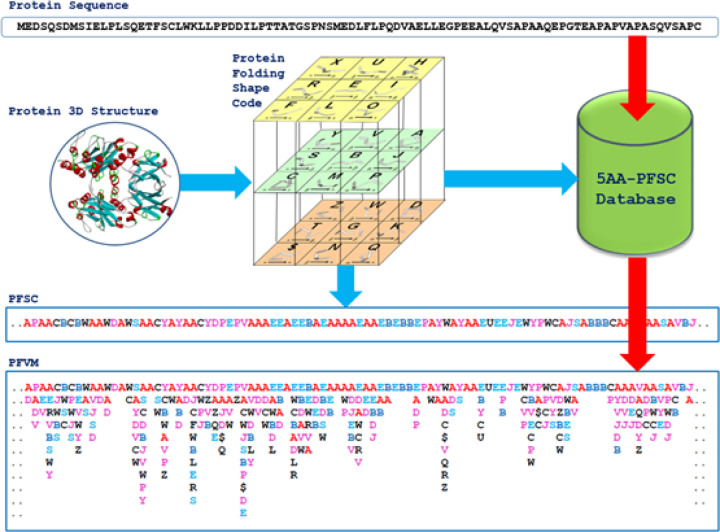
Protein structure fingerprint technology. The set of 27 protein folding shape code (PFSC) is presented in the cubic box. The blue arrows indicate how the complete conformation description with using PFSC is obtained from a protein 3D structure. The red arrows indicate how the comprehensive protein folding variations in the protein folding variation matrix (PFVM) are obtained from gene or protein sequence and expressed in PFSC description.

The RBD of the spike protein of SARS-CoV-2 binds to angiotensin-converting enzyme 2 (ACE2) receptors, serving as the entry point into human cells and causing the global coronavirus disease pandemic.^26^ Thus, analysis of mutations in the RBD of the SARS-CoV-2 spike protein is significant, as it helps to explain why SARS-CoV-2 has been more dramatically widespread than SARS-CoV and benefits vaccine and drug development. The structure variation in RBD fragment, as the affinitive interface with ACE2, is focused in this study, especially the evolution from SARS-CoV to SARS-CoV-2, and to the UK variant (lineage B.1.1.7) and India variant (lineage B.1.617.2). The complete sequences of the spike proteins were obtained from the Universal Protein Resource (UniProt) database,^27^ with UniProtKB P59594 for SARS-CoV (SPIKE_SARS) and UniProtKB P0DTC2 for SARS-CoV-2 (SPIKE_SARS2). Then, the mutational fragments of RBD sequences interfacing with ACE2 were aligned and displayed in Fig. 3-C and Fig. 3-D, which exposed the evolution of sequences from SARS-CoV to SARS-CoV-2, UK variant and India variant. The residues involving mutations are marked with bold font, in which SARS-CoV is black, SARS-CoV-2 blue, and UK variant and India variant red. Sequence alignment showed that the evolution of the RBD from SARS-CoV to SARS-CoV-2 involved the replacement of 9 residues; to the UK variant, two residues; and to the India variant, five residues.

The protein 3D structure of the complex of the spike protein and ACE2 is available in the PDB, and images of the interaction between the spike protein and ACE2 are displayed in Fig. 3. The protein structure with PDB ID 6ACG is the complex of the SARS-CoV spike protein and ACE2, which was deposited in July 2018; the structure with PDB ID 7A98 is the complex of the SARS-CoV-2 spike protein with ACE2, which was deposited in September 2020. The mutational residues of SARS-CoV-2 as well as the residues of ACE2 on the binding interface are marked by wire mesh to show the interpolated charged surface. It is apparent that most residues on the binding surface of ACE2 are negative charge and polar, except for K26 and K31 are positive charge. The binding surface of SARS-CoV has one residue, D480, with a negative charge facing negative residues on ACE2, and most residues of SARS-CoV are polar and without charge. After evolution from SARS-CoV to SARS-CoV-2, residue E484 near the positive residue K31 of ACE2 becomes negative, and the T501N mutation increases the polarity, which favors the interaction between SARS-CoV-2 and ACE2. All hydrogen bond (H-bond) interactions between the spike protein and ACE2 are listed in Fig. 3-A and Fig. 3-B. The distribution of H-bonds is different between SARS-CoV and SARS-CoV-2. For SARS-CoV, the residues on ACE2 involved in the H-bonds are K353, N330, Q325, Q42, Y41 and D38; for SARS-CoV-2, the residues on ACE2 involved in the H-bonds are Y83, Y41, H34, Q31 and Q24. This result indicated that the distribution of H-bonds shifted toward the N-terminus on SARS-CoV-2 compared with SARS-CoV. The change in the distribution of H-bonds is consistent with the influence of the residue 484 mutation on the spike protein from SARS-CoV to SARS-CoV-2.

The evolution from SARS-CoV to SARS-CoV-2, and to the UK variant and India variant enhanced the interaction of the spike protein with ACE2. The arrows in Fig. 3 indicate the mutational residues on the binding interface of the spike protein of SARS-CoV-2. Alteration to charge residues is an important factor in virus evolution. SARS-CoV does not have an effective charge residue on the interface with ACE2. In contrast, SARS-CoV-2 has residue E484 with a negative charge near the positive K31 of ACE2. In the UK variant, the E484K mutation reverses the charge of the residue from negative to positive and triggers a folding change, and K484 interacts with the nearby negative E23 on ACE2. In the recent variant from India, although the E484Q mutation changing from charge to polar, the N440K, L452R and T478K mutations changed to residues with positive charge. N440K changed from polar to positive charge and forwarded to negative residue E329 on ACE2; L452R changed from hydrophobic to positive charge and forwarded to negative residue D38 on ACE2; T478K changed from polar to positive charge and forwarded to negative residue E22 and E23 on ACE2. Also, it is noted that K417N mutation avoided the positive charge repulsion between K417 residue of spike protein and K31 residue of ACE2. These mutations in the Indian variant increase the affinity between the spike protein and ACE2. Overall, structural mutation analysis revealed that the evolution from SARS-CoV to SARS-CoV-2, and to the UK variant and India variant enhanced the spike protein interactions with ACE2, which helps the coronavirus to infiltrate into human cells and spread more easily, leading to the coronavirus disease pandemic.

## Discussion

The combination of sequence and protein structure fingerprint enhanced the study of mutations. In principle, sequence alignment is a useful means for studying mutations. First, it can be used to handle a large amount of data from databases with multiple sequence alignments for residue-by-residue investigation. Second, the protein structure fingerprint provides a complete description of protein folding without any gap, generating a unique sequence for alignment to study changes in folding conformation. With the protein structure fingerprint, the PFSC string as a complete folding description, which is acquired according to either the protein 3D structure or the PFVM, can cover regular secondary fragments and irregular tertiary fragments. Third, the alignment of PFSC strings is able to discover the folding structure difference caused by mutation. In addition, the combination of alignment of sequence with PFSC alphabetic string provides comprehensive analysis for mutation with integration of residue replacement and folding shape change. Moreover, the PFVM as folding variations, which is obtained directly according to protein sequence, reveals the fluctuation of the folding conformation along the sequence. It is significant that the protein structure fingerprint overcomes barriers in the study of the effects of mutations on protein structure when protein 3D structure data are absent. Thus, directly associating the protein sequence with the protein structure fingerprint is better to probe the mutations of SARS-CoV-2, which exhibits the changes on both physiochemical property and folding conformation, and provides more complete information understanding the variant in biological functions caused by mutations.

The mutations in fragment at the binding interface of the RBD of the SARS-CoV-2 spike protein interacting with ACE2 are critical for coronavirus epidemic spread. From SARS-CoV to SARS-CoV-2 (Fig. 3-A and Fig. 3-B), the interface at the RBD was involved with at least 9 residue mutations. Before SARS-CoV-2, the residues at the interface of SARS-CoV did not have apparent charge features, and most residues were polar. After evolution to SARS-CoV-2, residue E484 with a negative charge appears nearby positive charge residue K31 of ACE2, which is one of the factors making SARS-CoV-2 a more severe disease than SARS-CoV. The UK variant has 7 residue mutations (E484K, N501Y, A570D, P681H, T716I, S982A and D1118H) at the spike protein, but only E484K and N501Y are critical because of their positions at the binding interface of the RBD, which strengthen the interaction between SARS-CoV-2 and ACE2 to increase SARS-CoV-2 infectivity. The India variant has many mutations in the spike protein, but only K417N, N440K, L452R, T478K and E484Q impact the interaction with ACE2. Although the E484Q mutation reduced the charge feature of the residue, the N440K, L452R and T478K mutations generated three positive residues near the negative residues E329, D38, E22 and E23 in ACE2, and K417N mutation reduced repulsion. Thus, the mutations in the India variant enhanced the affinity between the spike protein and ACE2 and increased viral function. As viruses undergo genetic changes, some of these genetic changes can confer evolutionary advantages, and mutations of SARS-CoV-2 at the interface with ACE2 are especially critical. In the process of evolution, many mutations occurred at different positions in the spike protein and even on other proteins of SARS-CoV-2.^28^ Of course, some mutations may be neutral because they involve substitution of amino acids with physicochemical similarity; some mutations are missense because of substitution of amino acids with different physicochemical properties that change viral biological function. Understanding the structural variations caused by missense mutations in the protein epitope of SARS-CoV-2 is significant for antibody development. Thus, a protein structure fingerprint approach offers a better means to probe the mutations in SARS-CoV-2 and other viruses.

SARS-CoV-2 variant may be more transmissible than previously evolved ones, so understanding structural variations is important for drug development. The structural variations caused by mutations can provide lead information for vaccine and antibody research. With new mRNA vaccine technology, short-lived synthetic fragments of the RNA sequence of a virus is introduced into the human body, where they are taken up by dendritic cells, which use their own internal ribosomes to read the mRNA and produce viral antigen proteins. The synthetic mRNA fragment is a copy of the specific part of the viral RNA that carries the instructions to build the protein spike of SARS-CoV-2. Thus, the structural variations at the binding interface of the RBD of the SARS-CoV-2 spike protein provide an important reference for designing synthetic mRNA fragments. Developing a cocktail with multiple synthetic mRNA fragments according to the mutations in the fragment at the interface of the RBD of the spike protein may be a solution to continuously counter the evolution of SARS-CoV-2. Moreover, antibody engineering requires structural data related to spike protein mutations to design the product. Antibodies contain complementarity determining regions (CDRs) for a particular epitope on an antigen, which allow these two structures to bind together with precision. Mutations in the RBD of the SARS-CoV-2 spike protein provide significant structural information for CDR design and the production of effective antibodies. Thus, understanding the structural variations, particularly at the RBD of the spike protein of SARS-CoV-2, is significant for vaccine and antibody development.

## Conclusion

The alignment of the protein sequence and folding description reveals the structural variations caused by mutations of SARS-CoV-2. The protein structure fingerprint applies an alphabetical string to achieve a complete description of folding, which provides supplemental structural information for mutation study. The integration of changes in both physicochemical properties and folding features at the affinity interface of the RBD of the spike protein revealed how the coronavirus has gradually evolved in both structure and function and why SARS-CoV-2, the UK variant and Indian variant have led to more severe acute disease worldwide.

## Supplementary Material

Supplement 1
