# Supplementary material for: Exposing Structural Variations in SARS-CoV-2 Evolution
Source: Res Sq. 2021 Sep 13:rs.3.rs-800496. Preprint. [Version 1] doi: 10.21203/rs.3.rs-800496/v1 (PMC8452101; doi:10.21203/rs.3.rs-800496/v1)
Supplement: Supplement 1 [file 7808d8e7262f0f8a4f1abe85.docx]

**Table 1. The protein domain conservation and variation for sequences of SARS-CoV-like_Spike_S1_RBD subfamily of cd21477 in NCBI.** Red font indicates highly conserved, blue for less conserved and gray for unaligned as the threshold 3.5 for conservation alignment. Green background indicates amino acid differences between 6ACC-A and 6VSB-C.


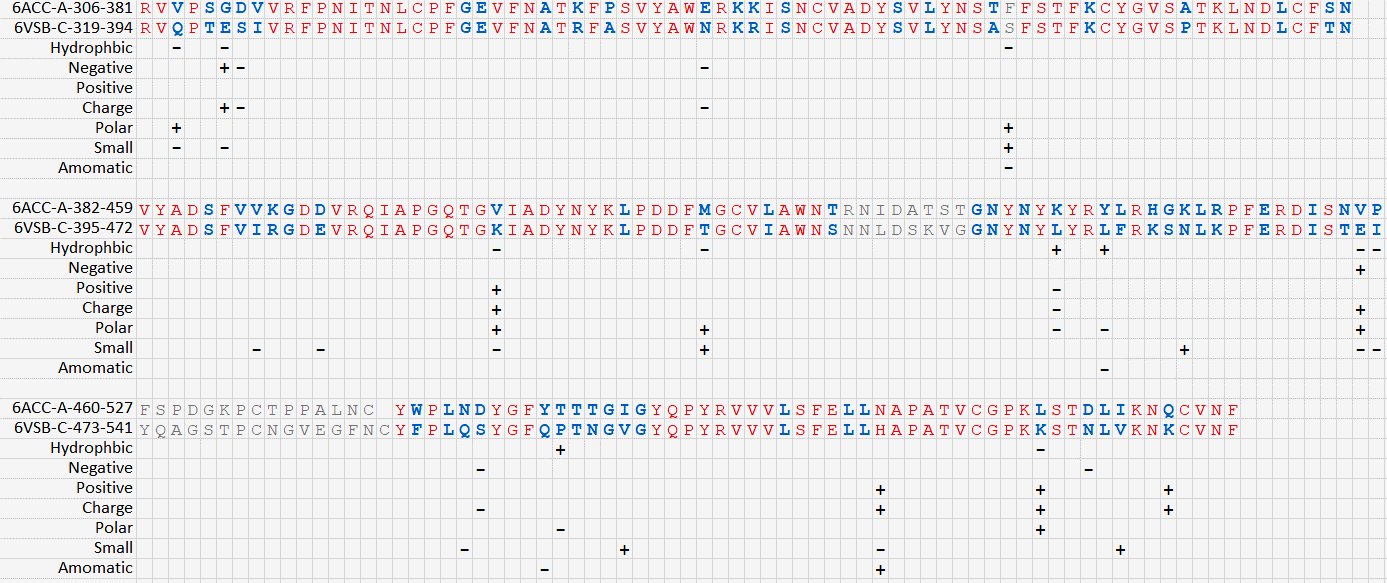


**Table 2. The change of physicochemical properties due to mutations from sequence of 6ACC to 6VSB.** Residues in red are highly conserved, blue are less conserved and gray are unaligned. The physicochemical properties are listed in the left column. The “+” sign indicates increase in the property after mutation; the “-” sign indicates a decrease in the property after mutation.


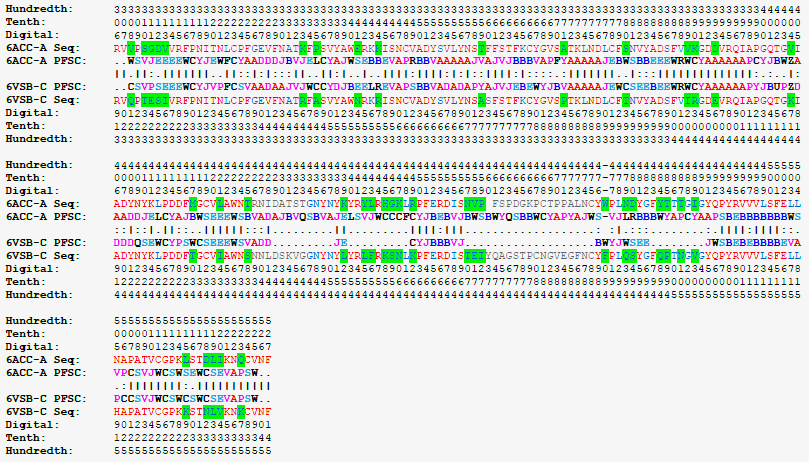


**Table 3. PFSC alignment between SARS-CoV (PDB 6ACC-A-306-527) and** [**SARS-CoV-2**](https://en.wikipedia.org/wiki/SARS-CoV-2) **(PDB 6VSB-C-319-541).** The rule of residue position and amino acid sequences are above or below the PFSC string. In the sequence, red font indicates highly conserved, blue indicates less conserved and gray indicates unaligned. Each PFSC letter represents the folding shape of a region of 5 amino acid residues. For PFSC, generally the red color indicates a typical alpha helix, pink indicates an alpha-like helix, blue indicates a typical beta strand, light blue indicates a beta-like strand, and black indicates an irregular fold. In the alignment, the local folding similarity and differences between PFSC strings are indicated; “|” indicates an identical folding shape, “:” indicates a similar folding shape, and “.” indicates dissimilar folding.


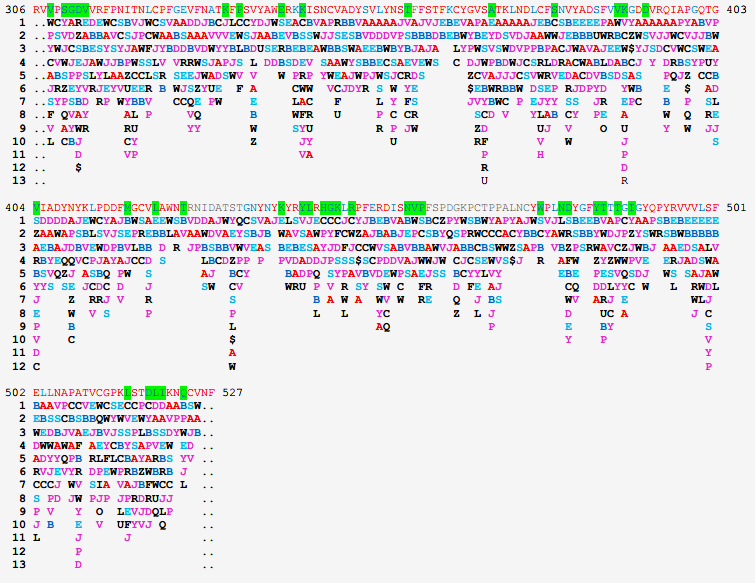


***********************************************************


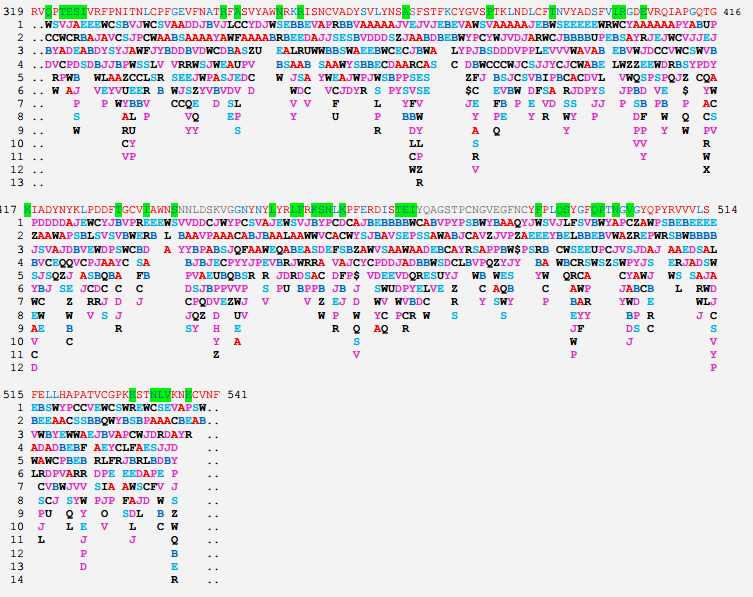


**Table 4. The protein folding variation matrix (PFVM).** The PFVM on top was obtained according to the sequence for PDB 6ACC-A-306-527 for SARS-CoV; the PFVM on bottom represents for PDB 6VSB-C-319-541 for [SARS-CoV-2](https://en.wikipedia.org/wiki/SARS-CoV-2). On each PFVM, the sequence is horizontally listed on above matrix, and the PFSC letters in each column represent the folding variations of continiouse 5 amino acid residues, and the most favored folding shapes are on top.


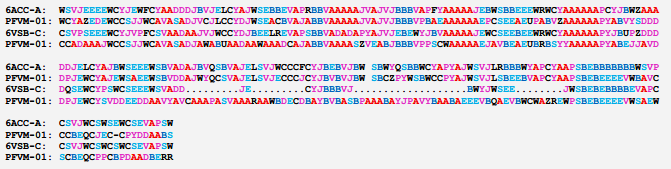


**Table 5. The alignment of PFSC strings of PDB 6ACC-A-306-527 for SARS-CoV and PDB 6VSB-C-319-541 for** [**SARS-CoV-2**](https://en.wikipedia.org/wiki/SARS-CoV-2)**, and the most likely folding conformation (PFVM-01) from PFVM.** The left column indicates the structure names. PFVM-01 is the PFSC string taken from the top row of each column in Table 4.


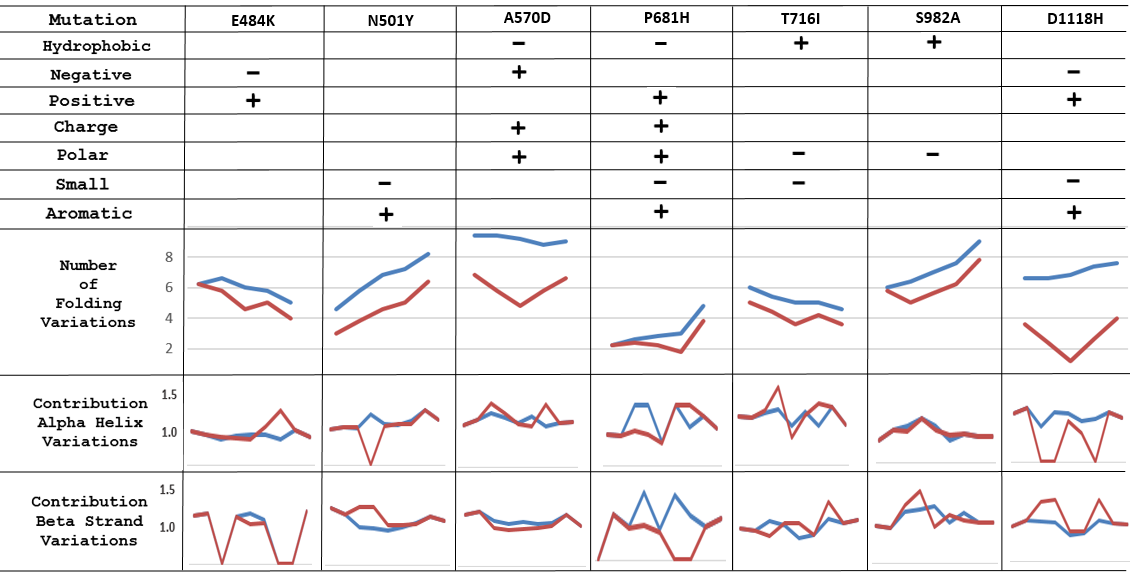


**Table 6. The variations of physiochemical properties and folding features for UK mutations.** GenBank QTJ15692.1 are taken as [SARS-CoV-2](https://en.wikipedia.org/wiki/SARS-CoV-2) reference. The potential changes of physiochemical properties are indicated on top rows, the “-” means a specific property decreased after mutation and the “+” property increased after mutation. The folding variations are listed at bottom three rows. The red curves represented the status after mutations; blue curves for status before mutations.


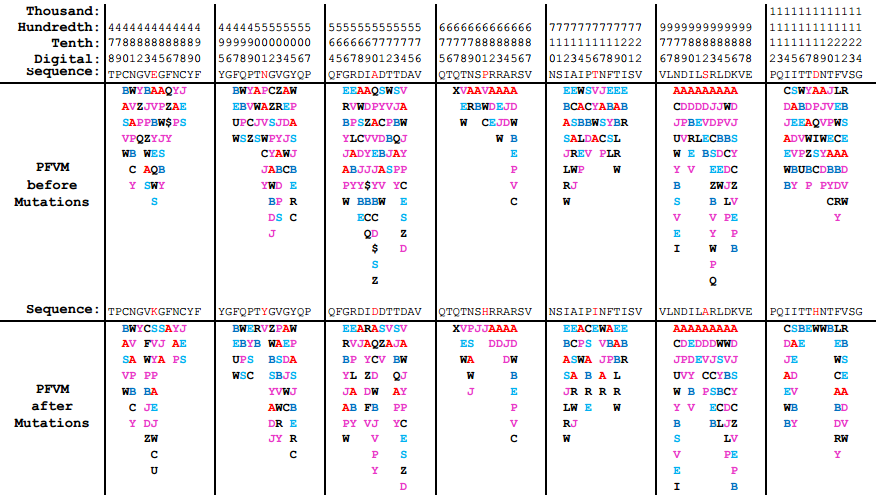


**Table 7. The sections of PFVM between** [**SARS-CoV-2**](https://en.wikipedia.org/wiki/SARS-CoV-2) **GenBank QTJ15692.1 and UK mutations.** The PFVM on top is for QTJ15692.1, and the PFVM below is for the UK variant (E484K, N501Y, A570D, P681H, T716I, S982A and D1118H). The rule for the residue position is at the top, the sequences are listed above the PFVM, and the mutated residues before and after mutation are shown in red. The PFVM consists of PFSCs. The PFSC letters in each column represent the folding variations of 5 continue amino acid residues, and the most favored folding shapes are on top.
